# Quality of teaching radiation oncology in Germany—where do we stand? Results from a 2019 survey performed by the working group “young DEGRO” of the German Society of Radiation Oncology

**DOI:** 10.1007/s00066-020-01623-x

**Published:** 2020-05-04

**Authors:** M. Oertel, P. Linde, M. Mäurer, D. F. Fleischmann, C. T. Dietzel, D. Krug

**Affiliations:** 1 Department of Radiation Oncology, University Hospital Muenster, Muenster, Germany; 2 Department of Radiation Oncology and Cyberknife Center, University Hospital of Cologne, Cologne, Germany; 3 Department of Radiation Oncology, Jena University Hospital, Jena, Germany; 4 Department of Radiation Oncology, University Hospital, LMU Munich, Munich, Germany; 5 partner site Munich, German Cancer Consortium (DKTK), Munich, Germany; 6 German Cancer Research Center (DKFZ), Heidelberg, Germany; 7 Department of Radiation Oncology, University Hospital Halle (Saale), Halle (Saale), Germany; 8 Department of Radiation Oncology, University Hospital Schleswig-Holstein, Campus Kiel, Kiel, Germany

**Keywords:** medical education, radiooncology teaching, innovative teaching, competence-based learning, NKLM

## Abstract

**Purpose:**

Medical students’ knowledge of radiation oncology (RO) is of increasing importance with a rising prevalence of malignancies. However, RO teaching in medical schools is heterogeneous and has not been analyzed at a federal level yet. Therefore, the following survey aims to provide a national overview of RO teaching in Germany.

**Methods:**

A questionnaire containing multiple-choice and free-text questions covering the extent and topics of RO teaching was sent to RO departments of all university hospitals in Germany and was answered by the heads of department/main lecturers.

**Results:**

24/35 (68.6%) RO departments returned completed forms. Most faculties employ lectures (91.7%), seminars (87.5%), and practical/bedside training (75.0%), whereas training in radiation biology and medical physics are rare (25% and 33.3%, respectively). Main topics covered are general RO (100%), radiation biology (91.7%), and side effects (87.5%). Regarding RO techniques and concepts, image-guided and intensity-modulated radiotherapy are taught at all faculties, followed by palliative and stereotactic techniques (87.5% each). Notably, all departments offered at least a partial rotation in RO in conjunction with radiology and/or nuclear medicine departments in the last year of medical school, while only 70.8% provided a complete rotation in RO. In addition, 57.1% of the departments have taken measures concerning the upcoming National Competence-Based Learning Objectives Catalogue (NKLM) for medical education.

**Conclusion:**

RO plays an integral but underrepresented role in clinical medical education in Germany, but faces new challenges in the development of practical and competence-based education, which will require further innovative and interdisciplinary concepts.

**Electronic supplementary material:**

The online version of this article (10.1007/s00066-020-01623-x) contains supplementary material, which is available to authorized users.

## Introduction

Increasing numbers of patients are diagnosed with cancer each year, reaching an annual incidence of over 450,000 patients in Germany [1]. With around 50% of oncological patients having an indication for radiation therapy (RT) during their course of disease [2, 3], knowledge of radiation oncology (RO) has cardinal importance for both oncological disciplines as well as for general practitioners to provide patients with adequate counsel. However, there are common misbeliefs about RO and its treatment spectrum, which persist until the end of medical school [4], demanding an improvement in teaching. Innovative concepts such as internships or interdisciplinary classes could be of additional value [4, 5], but are not obligatory across Germany. At the same time, the introduction of the new German National Competence-Based Learning Objectives Catalogue (NKLM) [6] calls for interdisciplinary and practice-orientated teaching formats. Despite the need for reformed and innovative teaching, a survey by a working group of the German Society of RO (young DEGRO) revealed that only around 20% of young radiation oncologists are able to perform teaching activities within the regular working hours [7]. The obvious discrepancy between the demand for restructured curricula and the uncertainties concerning formal requirements prompted our working group to perform a survey on the current situation of RO teaching in Germany.

The aim of this study is to describe the state-of-the-art of RO medical education in Germany, to identify potential fields of further development, and to delineate future challenges ahead.

## Materials and methods

A detailed questionnaire assessing extent and topics of RO teaching was designed by the working group of the German Society for RO (young DEGRO) in a peer-review process. The questionnaire contained both open and multiple-choice questions and was sent in written form and/or electronically to all academic RO departments at university hospitals in Germany (see Supplementary 1 for full questionnaire). Answers were analyzed using SurveyMonkey (SurveyMonkey, Dublin, Ireland) and Microsoft Excel for Mac, Version 16.30 (Microsoft Corporation, Redmond, Washington, USA).

## Results

**Fig. 1:**
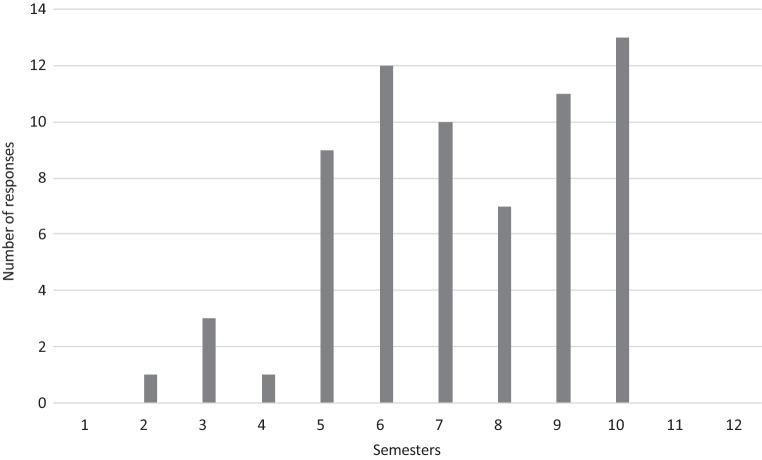
Responses to the question “During which semesters does radiation oncology teaching take place?” Multiple answers were allowed

**Fig. 2:**
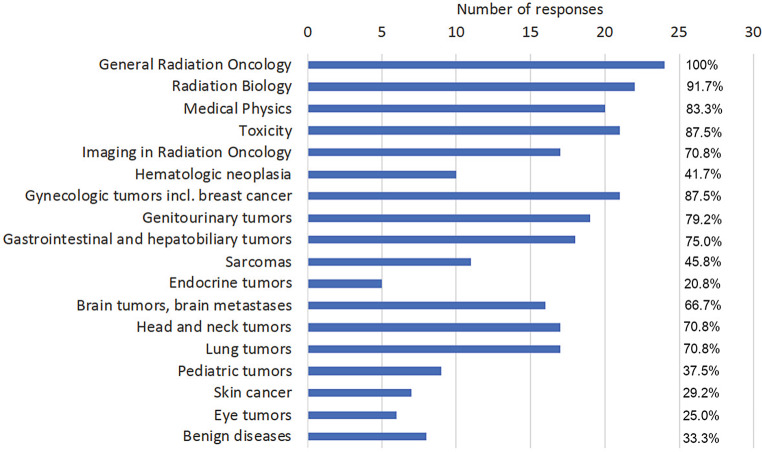
Responses to the question “Which topics are addressed during radiation oncology teaching?” Multiple answers were allowed

**Fig. 3:**
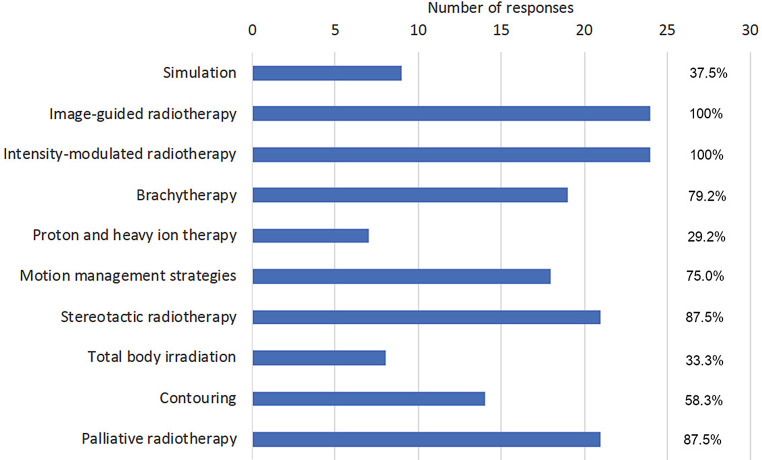
Responses to the question “Which techniques and concepts are included in radiation oncology teaching?” Multiple answers were allowed

Complete questionnaires were received from 24 out of 35 university hospitals (68.6%). In some cases, RO teaching starts in pre-clinical semesters (second semester: 1, third semester: 3), but is found mostly in the second half of medical education during clinical semesters (e.g., fifth semester 37.5%, sixth semester 50%, seventh semester 41.7%, eighth semester 29.2%, ninth semester 45.8%, tenth semester 54.2%; Fig. 1). Teaching is performed by heads of department/directors (100%), consultants (95.8%), residents (91.7%), and external lecturers (58.3%). The predominant teaching formats are lectures (91.7%), seminars (87.5%), and practical/bedside training (75.0%), whereas training in radiation biology and medical physics is less frequent (25% and 33.3%, respectively). The main topics covered in RO teaching are general RO (100%), radiation biology (91.7%), side effects (87.5%), and radiation physics (83.3%; Fig. 2). Concerning the different organ systems, gynecological (87.5%), urogenital (79.2%), gastrointestinal (75%), head and neck, and lung tumors (70.8% each, respectively) ranked highest. Interdisciplinary teaching formats have been established at 87.5% of faculties, whereas a longitudinal oncological curriculum is only present in 41.7% of the faculties. Multidisciplinary concepts incorporate gastroenterology (81%), gynecology, urology (76.2% each), brain tumors/cerebral metastases, and lung tumors (57.1% each). Main RO techniques and concepts taught are image-guided and intensity-modulated RT (100% each), stereotactic and palliative techniques (87.5% each), and brachytherapy (79.2%; Fig. 3). There is a possibility for an internship during the final practical year in RO at all faculties, but only 70.8% offer a complete rotation (4 months). Elective teaching courses are offered at 75% of faculties covering additional subjects of RO. Essentially, 57.1% of radiation oncology faculties have taken measures addressing implementation of the *Masterplan Medizinstudium 2020* and the accompanying NKLM for medical education.

## Discussion

The presented survey describes the current situation of RO teaching in Germany and displays a variety of individual curricula with heterogeneity regarding extent, topics, and onset.

Qualified teaching is of pivotal importance and a major factor for RO resident recruitment [8]. Additionally, RO education is highly relevant to medical students to provide oncological knowledge for later residency. However, the survey demonstrates heterogeneity in the extent and content of RO teaching throughout Germany. Some medical faculties perform seminars and lectures in one semester only or have no obligatory lecture at all, whereas others have developed extensive curricula with representation of RO in all clinical semesters.

Consequently, the spectrum of topics differs greatly. Whereas “RO in general/introductory lectures” are always held, only 70% of curricula include “lung,” “head and neck,” or “imaging in radiation oncology.” “Hematology,” “skin,” “pediatric,” or “endocrinology” received percentages below 45%. It is particularly striking that only 1/3 of medical faculties include benign diseases in their curricula. Interestingly, a comparable survey in 19 European countries, involving 32 academic institutions, showed a similar thematic selection with large heterogeneities in teaching extent (2–60 h of RO teaching), the responding German institution being in the lower third [9]. Another analysis conducted in Australia and New Zealand unveiled a worse situation, with at least 50% of faculties, including 81% of students, not receiving training in basic principles of RO [10]. In summary, this minor role in the current curriculum does not adequately reflect the cardinal importance of the field in oncological patient care.

A disparity in knowledge of different entities persists even in RO residency, as displayed by a survey of the young DEGRO [11]. The implication may be discussed controversially: regarding the fact that many medical students will not choose oncological residencies, Haagedorn and de Vries argued for a change in teaching practice addressing the general practitioners’ need for basic oncology education rather than focusing on details of biology and treatment [12].

What is the amount of RO teaching that is essential for every medical student? Which tumor entities should be mandatorily included in the curriculum of medical schools? These questions, amongst others, remain debatable and are currently being discussed by a new working group of the DEGRO (see below). It can be summarized that there is increasing consensus that a basic RO education for medical students should at least comprise the main oncological entities (breast, prostate, lung, head and neck, metastases in different locations …) in Germany, as indicated by the cancer report of the Robert Koch Institute [1]. Moreover, it is our strong belief that practical training in oncological anamnesis, empathic counseling and guidance, and focused physical examination should be integrated.

In contrast to the vivid discussions on the overall scope of basic RO education during medical school, the teaching of RO techniques and concepts appears homogenous, with all faculties imparting the principles of modern image-guided and intensity-modulated RT. Only a minority of medical schools in Germany (<30%) present particle therapy or heavy-ion therapy, which may be due to the limited distribution of the respective treatment facilities.

The abovementioned heterogeneity may be attributed to the different concepts of medical education. While the “regular” course teaches RO within the module “imaging procedures, radiation treatment, and radiation protection” together with radiology and nuclear medicine, the reformed or model curriculum (e.g., model curricula in Aachen, Berlin, Hannover, and Heidelberg and reformed curricula in Cologne and Muenster) aims at a concept-based education. Accordingly, organ-specific modules are established in which interconnectivity between the oncological disciplines takes center stage.

These challenges are being met by the DEGRO by the formation of a new working group focusing on the redefinition of learning objectives and key abilities in light of the new *Masterplan Medizinstudium 2020*. Its main goal will be the establishment of a national model curriculum for RO in medical education, serving as a blueprint for the individual faculties. A comparable schedule already exists for residents and will likely be refined within the near future [13]. Additionally, a reciprocal dialogue between RO and other disciplines, like surgery and internal medicine et cetera, will ultimately lead to a new interdisciplinary clinical schedule, strengthening cardinal aspects of RO.

The survey is restricted due to the rate of incomplete responses (2/3 responders), with a possible selection bias, as faculties more involved in teaching might have been more responsive to the survey. This might lead to a bias in the results and an underestimation of potential problems in the field of radiation oncology teaching. Furthermore, the evaluation and opinions offered for this survey may not represent a consensus of the whole teaching staff at the respective sites, as the survey was sent to the heads of the departments. Thus, the survey has to be seen as a punctual observation, which nevertheless may give suggestions for further improvements of RO teaching and implementation of new teaching concepts.

Recently, the German government introduced the *Masterplan Medizinstudium 2020*, summarizing a package of measures for structural reorganization [14]. Its main goals include the vertical integration between clinical and undergraduate subjects, the deepening and consolidation of scientific knowledge, as well as the introduction of competence-based learning [14]. Therefore, it corresponds with global trends towards competence-based medical education in accordance with today’s medical students’ desire for personalized, interconnected, and team-based learning [15, 16].

The formal requirements, as listed in the NKLM [6], call upon medical faculties and their teaching staff (especially habilitated members) to thoroughly review their timetables, i.e., to change learning objectives from pure knowledge acquisition to practical application. Nevertheless, only a slight majority of faculties in the presented survey (57.1%) have already taken measures concerning the NKLM, clearly calling for further action. Competence-based teaching may favor practical formats like bedside teaching or seminars over lectures, but lectures are still the predominant teaching format at present. Additionally, interdisciplinary formats, which cover different subjects and at the same time bridge clinical and undergraduate education, may gain even more importance in the future by providing an introductory multimodal overview [16–18]. A recently published example is the course “Anatomy and Imaging” at the University of Muenster transferring anatomy knowledge to its clinical application, like in RO [5, 20, 21].

The strategy of early integration of RO teaching may also be suitable to avoid misbeliefs in RO. A multi-institutional survey in the US showed deficiencies in knowledge concerning RO indications, toxicity, and techniques affecting first-year and fourth-year medical students [4]. An RO rotation could improve responses in all categories [4]. Considering this finding, the number of faculties offering a complete RO rotation (70.8%) for final-year medical students should be increased. It is noteworthy that structured didactic sessions for clerkships could improve both students’ knowledge and interest in the field, but also lead to better evaluation results [22–25], and should therefore be considered for the RO rotations in Germany.

Future efforts of the RO community should focus on an appropriate as well as interesting presentation of the discipline to medical students to avoid misconceptions, but also to attract potential RO residents. This topic has been addressed by several publications of our working group demonstrating the spectrum of RT clinical routine and research [26–28]. Corresponding to this, free-text comments within the current analysis revealed a spectrum of elective teaching formats offered to interested students, covering palliative care, pediatric radiation oncology, neuro-oncology, and more. This demonstrates the creativity of the RO teaching community in Germany as well as the vivid interest in implementing state-of-the-art teaching formats.

## Conclusion

The current survey displays heterogenous curricula across Germany and emphasizes the need for a shift towards interdisciplinary and competence-based teaching formats. Shaping and defining a nationwide standardized curriculum with key abilities and knowledge of RO will be one of the key challenges for the DEGRO and the young DEGRO working group.

## Questionnaire

Umfrage der AG jDEGRO der DEGRO – Team Weiterbildung
