# Supplementary material for: Quality of teaching radiation oncology in Germany—where do we stand? Results from a 2019 survey performed by the working group “young DEGRO” of the German Society of Radiation Oncology
Source: Strahlenther Onkol. 2020 May 4;196(8):699–704. doi: 10.1007/s00066-020-01623-x (PMC7385026; doi:10.1007/s00066-020-01623-x)
Supplement: Supplementary file 1 — Umfrage der AG jDEGRO der DEGRO – Team Weiterbildung [file 66_2020_1623_MOESM1_ESM.docx]

|  | 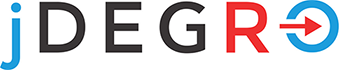 |
| --- | --- |
|  | AG junge DEGRO  Deutsche Gesellschaft für Radioonkologie e.V. Geschäftsstelle: Reinhardtstr. 47, 10117 Berlin |

Umfrage der AG jDEGRO der DEGRO – Team Weiterbildung

In welchen Semestern findet strahlentherapeutische Lehre statt (Gesamtsemester des Medizinstudiums)

| 1 | 2 | 3 | 4 | 5 | 6 | 7 | 8 | 9 | 10 |
| --- | --- | --- | --- | --- | --- | --- | --- | --- | --- |
|  |  |  |  |  |  |  |  |  |  |

Welche Formate existieren an Ihrer Fakultät?

- Vorlesungen
- Lehrvisite/Praktikum am Krankenbett
- Seminare
- Strahlenbiologisches Praktikum
- Physikalisches Praktikum
- Sonstige: _____________

Welche Entitäten/Lehrinhalte werden im Strahlentherapie-Lehrplan abgedeckt?

- Strahlentherapie allgemein/Einführung
- Strahlenbiologie
- Strahlenphysik
- Nebenwirkungen
- Bildgebung in der Strahlentherapie
- Hämatologische Erkrankungen
- Gynäkologische Erkrankungen
- Urologische Erkrankungen
- Gastrointestinaltrakt, hepatobiliäres System
- Sarkome, Knochentumore
- Endokrine Tumore
- Hirntumore/cerebrale Metastasen
- HNO-Tumore
- Lungentumore
- Pädiatrische Neoplasien
- Hauttumore
- Ophtalmologische Tumore
- Benigne Erkrankungen
- Andere:

Werden interdisziplinäre onkologische Veranstaltungen (z.B. zusammen mit Chirurgie, Hämatologie/Onkologie, Gynäkologie, Urologie etc.) angeboten?

- Ja, in Form von _________________
- Nein

Welche Entitäten werden im Rahmen dieser integrativen Formate abgedeckt?

- Hämatologische Erkrankungen
- Gynäkologische Erkrankungen
- Urologische Erkrankungen
- Gastrointestinaltrakt, hepatobiliäres System
- Sarkome, Knochentumore
- Endokrine Tumore
- Hirntumore/cerebrale Metastasen
- HNO-Tumore
- Lungentumore
- Pädiatrische Neoplasien
- Hauttumore
- Ophtalmologische Tumore
- Benigne Erkrankungen
- Andere:

Gibt es ein eigenständiges longitudinales onkologisches Curriculum?

- Ja
- Nein

Wenn ja, ist die Strahlentherapie an dem eigenständigen longitudinalen onkologischen Curriculum beteiligt?

- Ja
- Nein

Welche strahlentherapeutischen Techniken werden im Lehrplan vermittelt?

- Simulation
- Bildgeführte Strahlentherapie (IGRT)
- Intensitätsmodulierte Strahlentherapie (IMRT)
- Brachytherapie
- Protonen- und Schwerionentherapie
- Berücksichtigung von Organbewegungen (4D-CT, Gating, Tracking)
- Stereotaxie
- Ganzkörperbestrahlung (TBI)
- Konturierung
- Palliativmedizin

Existiert die Möglichkeit, Abschnitte des Praktischen Jahres in Ihrer Klinik zu absolvieren?

- Ja, vollständiges Tertial/Quartal
- Ja, in Rotation mit __________________
- Nein

Bietet Ihre Klinik zusätzliche Lehrinhalte über Wahlfächer an?

- Ja
- Nein

Wenn ja, welche sind dies (Titel und kurze Beschreibung)

Welche zusätzlichen strahlentherapeutischen Lehrinhalte würden Sie sich im Stundenplan Ihrer Fakultät wünschen?

Welches Verbesserungspotential sehen Sie (allgemein) im Hinblick auf die strahlentherapeutische studentische Ausbildung?

Sind an Ihrer Fakultät/an Ihrer Einrichtung bereits Vorbereitungsmaßnahmen auf die Umsetzung des NKLM getroffen worden?

- Ja, und zwar ____________________________
- Nein

Wer ist an der Durchführung der strahlentherapeutischen Lehre beteiligt?

- Chef-/Oberärzte
- Fachärzte
- Assistenzärzte
- Externe Habilitierte
